# Thermodynamic studies and optimization of pectinase produced by soil-isolated *Aspergillus foetidus* and its application in the textile industry

**DOI:** 10.1038/s41598-026-57508-z

**Published:** 2026-06-24

**Authors:** Sara H. Mansour, Eman F. Ahmed, Naser G. Al-Balakocy, Elsayed A. Elsayed

**Affiliations:** 1 Chemistry of Natural and Microbial Products Department, National Research Centre, Dokki, Cairo Egypt; 2 Protein & Manmade Fibers Department, National Research Centre, Dokki, Cairo, Egypt

**Keywords:** Pectinase, *Aspergillus foetidus*, Optimization, Kinetic study, PET/C fabric, Biochemistry, Biological techniques, Biotechnology, Microbiology

## Abstract

The current study aimed to produce pectinase enzyme from locally isolated fungus and to improve its industrial applicatbility as an eco-friendly alternative to chemical agents traditionally used in textile industries. The most active fungal isolate was genetically identified as *Aspergillus foetidus* (NR_163668.1). Submerged fermentation allowed maximal pectinase production yield of 138 U/mg at pH 4.5 and 37 °C after 5 days of incubation. Among six agro-industrial substrates evaluated; 4% wheat bran, supplemented with 1% yeast extract and 0.2% EDTA resulted in maximal enzyme production. The produced pectinase showed higher stability at acidic conditions after 15 and 30 min. Thermal kinetic parameters, including half-life (t₁/₂), enthalpy change (ΔH), Gibbs free energy (ΔG), and entropy change (ΔS), were determined, confirming high thermal stability. These properties support its potential biocatalytic application in textile industries. To confirm the feasibility of the produced pectinase enzyme in textile industry, optimized conditions were applied to treat soured polyester/cotton (PET/C) blend fabric. The reaction was performed at 50 °C and pH of 5.0 for 40 min. These conditions led to the removal of the sizing agent (starch) with a total weight loss of PET/C fabric of approximately 4%.

## Introduction

 Enzymes are biological compounds present in the cells of macro- and microorganisms. They are very specific to catalyze cellular chemical reactions without altering the chemical structure by the end of the reaction. Enzymes have a high specificity in the catalytic activity of different reactions. Due to their advantages, enzymes found different applications in pharmaceutical, and chemical industries^1^. Microorganisms such as bacteria, fungi and yeasts are the main sources for industrial enzymes due to their higher growth and cell division rates, short life cycle, as well as ease of genetic manipulation. Among all industrial enzymes, pectinases are the most important in degrading pectin which is a long polysaccharide chain found in the cell walls of plants. The degradation reaction releases d-glucuronic acid as an end product^2,3^. The vast majority of industrial pectinases were produced from fungal organisms^4^. Pectin degrading enzymes were used in juice industry to breakdown pectin long chains and decrease the viscosity and improve the clarification of the fruit juice.

Treatment with pectinase enzymes results in the extension of plant cell walls, and soften the plant tissues at maturation stage. Therefore, they have an ecological importance in decomposition and recycling processes of plant wastes^5^. Pectinolytic fungi produce pectinolytic enzymes that degrade pectin. The most efficient and popular fungi are *Penicillium restrictum*,* Aspergillus niger*,* Mucor piriformis*, *Trichoderma viride*, *Yarrowia lipolytica* and *Aspergillus awamori*^3^. Other potent producers of pectinase are *Tetracoccosporium* species which showed pectinolytic activity as 20 mm clearance zone around their colonies^6^.

To avoid the breakage of cotton or blend fiber threads during weaving process, an adhesive substance, the “size” is generally used to coat the warp. Traditionally, weaving industry applies various compounds to size fabrics, however, starch and its derivatives are recommended for applicaton. Depending on on the source of the raw materials used, starch is readily available as cheap^7^. During preparation of dyeing and finishing steps and after being weaved, the fabric must be treated again to remove the sizing agent; desizing process. This strep is performed using chemical acids, alkalis or oxidizing agents. However, due to the chemical reaction nature, this removal process does not insure total removal of starch. Accordingly, the dyeing process has several disadvantages. Moreover, treated cotton fibers will degrade leading to loss of natural cotton soft feel. Therefore, its is highly recommended to use various microbial degrading enzymes due to their higher efficiency and specific action. Amylases have been widely applied as desizing microbial enzymes for starch removal^8^.

During a traditional scouring process, caustic soda and sodium hydroxide are used to treat fabrics at 70–90 °C. However, strong alkaline agents can severly affect the resultant fabric weight (g/m^2^), and also will lead to difficult environmental pollution situations. Therefore, microbial enzymes are used to avoid impacting fabric nature or the environment itself. Starch hydrolysis using microbial pectinases will allow complete removal of starch, which will finally lead to good water absorbance while avoiding the negative impacts of cellulose destruction, the so-called “bio-scouring”. The cotton cell wall’s pectins, which bind waxes, oils, and other contaminants and make their removal easier, are hydrolyzed by pectinase^9–13^. Better wetting and penetration qualities are provided by the fabric, which facilitates the bleaching process and improves dye uptake.

This study was conducted to isolate new potent pectinolytic strains from Egyptian garden soil. The aim was extended to optimize the composition of the production medium as well as the cultivation conditions for maximal enzyme production. The produced enzyme was then partially purified, characterized, and kinetically investigated. Furthermore, the industrial application of the produced pectinase in bio-scouring of PET/C fabric was investigated. Pectinase breaks down the pectin in the cotton and thus assists in the removal of waxes, oils and other impurities. PET/C blend fabric gives better wetting and penetration properties, making subsequent industrial process easier and allows for much better dyeing and finishing wet operations.

## Methods

### Fabric

Scoured polyester/cotton (50:50) blend (PET/C) (236 gr/m^2^) woven fabrics were used throughout this work. All types of used fabrics were provided by local textile industries.

### Samples collection and maintenance

Soil samples were collected from author′s private garden in Obour City (30.19395, 31.46551), Qaluibya Government, Egypt. Specimens were taken from the area around different fruit trees (Mango, pomegranate, Lemon, pears and apricot). The samples were maintained in clean bags in the fridge.

### Buffers and Solutions Used for Isolation and Identification of DNA

For DNA isolation and purification, 5X Tris-Borate-EDTA Buffer (TBE), pH 8, was prepared by dissolving 0.29 g Na-EDTA, 5.41 g Tris-HCl, pH 8, and 2.75 g boric acid in 100 ml H_2_O. 10 µg/ml ethidium bromide stock solution was used. 5X-Sample loading dye was prepared by mixing 2 ml of 0.5 M Na-EDTA, pH 8.0, 5 ml of 100% glycerol, 0.75 ml of 2% bromophenol blue, 2% xylene cyanol and 1.5 ml H_2_O. For gel preparation, 100 g agarose was dissolved in 100 ml 1X-TBE and 5 µl of 1% ethidium bromide solution.

### Screening and isolation of pectinase producer’s fungi

Different pectinase producing fungal strains were isolated according to standard isolation protocols^14^. Definite weight (10 g) of the sample was suspended in 50 ml distilled water and shaken for 5 min. Afterwards, 100 µL of the suspension was added to a selective pectin production medium in Petri dishes and incubated for 7 days at 28 °C. The cultivation medium was composed of (g/100 ml): NH_4_PO_4_, 0.3; KH_2_PO_4_, 0.2; K_2_HPO_4_, 0.3; MgSO_4_; Agar, 2.5 and Pectin, 1. The obtained colonies in the petri dishes were exposed to Iodine vapor and the inhibition zones were measured.

### Submerged fermentation medium for pectin degrading enzyme

Production of pectinase was achieved by submerged fermentation^15^. 2.5 ml Spore suspension (4⋅10^8^ spores/ml) of the isolated fungal strains was inoculated in 50 ml potato dextrose (PD) broth medium for 2 days at 28 °C. Afterwards, 2.5 ml of the growing cells was used to cultivate flasks containing 50 ml pectinase production medium (g/l; Pectin, 10; KCl, 1.0; MgSO_4_, 0.5; Yeast extract, 0.5; K_2_HPO_4_, 1.0).

### Agricultural waste fermentation medium

For investigating different agricultural waste, different pectinase producing isolates were grown in medium contained (g/l): KCl, 1.0; Yeast extract, 1.0 and 10.0 g of different agricultural wastes evaluated; these included wheat bran, rice bran, banana peels, citrus peels and sugar cane bagasse. Each waste was added as a sole carbon source in the fermentation medium.

### Optimization of cultivation conditions

Different cultivation conditions were evaluated for their optimal effects on pectinase production^16^. These conditions included incubation period, inoculum size as well as cultivation temperature and pH.

### Optimization of production medium

The composition of the production medium was optimized by varying the concentrations of different medium components including wheat bran concentration, organic nitrogen composition, as well as salt additives.

### Partial precipitation of Pectinase enzyme

Purification of the enzyme was partially performed by increasing the volumes of Acetone (30, 40, 50, 60, 70, 80, and 90%)^17^. Each volume was added slowly to an ice cold-enzyme solution. At the end of each level; the whole solution was centrifuged and the partially purified enzyme was dried and weighed. The activity of the pectinase enzyme at each solvent concentration was determined.

### Pectinase activity and protein content determination

The activity of pectinase produced was determined using 3.5- dinitrosalicylic acid (DNS) method^14^. 0.1 ml of the reaction mixture containing 0.5 ml pectin as a substrate dissolved in citrate buffer (0.05 M pH 4.4) and 0.5 ml diluted enzyme (0.2 g in 2 ml citrate buffer pH 4.4) was incubated at 30 °C for 30 min in a water bath. Finally, DNS reagent was added and the measurement was read at 550 nm. Protein content was investigated according to Lowery et al.^18^.

### Characterization of partially purified enzyme

#### Reaction pH and pH stability

The effect of pH and pH stability of the partially purified pectinase enzyme were determined^19^. 10 mg of the partially purified enzyme were dissolved in 5 ml buffer solution with pH range from 3.0 to 9.0. The test was applied and the activity of the enzyme was assessed. In pH stability experiment, the partially purified enzyme (10 mg) was dissolved in the buffer solution (5 ml) at different pH values ranging from 3.0 to 9.0 for 15 and 30 min. Afterwards, the enzyme was incubated with the substrate, and the data of the relative activity were drawn.

### Incubation temperature and thermal stability

To determine the effect of incubation temperature on the activity of the enzyme^20^, equal volumes of the enzyme and the substrate were incubated at increasing levels of temperatures (30, 40, 50, and 60 °C). In thermal stability experiment, the enzyme (0.5 ml) was exposed to different temperatures (40, 50, 60, 70 and 75 °C) for 15, 30, 45 and 60 min before being incubated with the substrate at 30 °C for 30 min.

### Effect of different concentrations of pectin on enzyme activity and the determination of V_max_ and K_m_^21^

Definite concentration of the enzyme was incubated with different concentrations of the substrate (0.02, 0.04, 0.06, 0.08, 0.1, and 0.12 mM). The relative activity was determined and V_max_ and K_m_ were calculated. The Line Weaver-Burk plot (double reciprocal) method was used to obtain the Michaelis-Menten kinetic models adequate for the description of the hydrolysis of pectin by the pectinase. Apparent K_m_ and V_max_ of pectinase enzyme was determined by plotting 1/[S] against 1/[V], respectively. [S]/V_o_ = 1/V_max_ * [S] + K_m_/V_max_ Where, [S] is the substrate concentration (pectin), V_o_ is the initial enzyme velocity, V_max_ is the maximum enzyme velocity, and K_m_ is the Michaelis constant and is defined only in experimental terms and equals the value of [S] at which Vo equals 1/2 V_max_.

### Molecular identification of fungal strain

#### Isolation of genomic DNA

Fungal isolates were grown in Potato Dextrose broth for 72 h and were harvested by centrifugation at 11,000 g for 5 min. Genomic DNA was extracted using Gene JET Genomic DNA purification kit (Thermo Scientific, Lithuania) according to the manufacturer’s recommendations.

### PCR amplification of ITS gene

Amplification was done using primer ITS1F (5′- CTTGGTCA TTTAG A GGAAGTAA − 3′) and reverse primer ITS4R (5′- TCCTCCGCTTATTG ATATGC − 3′). The PCR mixture was carried out in a volume of 50 µl, containing 22 µl of MQ, 25 µl of Dream Taq Green DNA Polymerase (Thermo Fisher Scientific, USA), 1 µl of each forward and reverse primer (10 µmol/l), and 1 µl of the template. The PCR amplification conditions were 4 min of preheating at 95 °C, 30 s denaturation at 95 °C, 45 s of primer annealing at 52 °C, 1 min extension step at 72 °C, and post cycling extension of 10 min at 72 °C for 35 cycles. The reactions were carried out in a thermal cycler (Applied Biosystem Thermal Cycler, USA).

### Sequence alignment, phylogenetic analysis, and bioinformatics analysis

Amplified PCR product was purified and sequenced at Macro gene, Korea. Raw data of sequencing were edited (connoting and peak chromatogram verification) using the Finch T.V 1.4.0 program. Analysis of ITS sequences of strain was performed using the BLAST (N) program of the National Center of Biotechnology Information (NCBI) (Rockville Pike, Bethesda MD, USA)^22^. Multiple sequence alignment was done using the Clustal W 2.1 program. The phylogenetic trees were constructed using neighbor joining method by MEGA 11^23^.

### PET/C fabrics activated by pectinase

The treatment of PET/C blended fabrics with pectinase was carried out using a high-temperature high-pressure laboratory dyeing machine^24^. The required volumes of pectinase were placed in stainless-steel bowls (1 and 3%). Then, fabrics samples were immersed in the solutions and the pH was adjusted to 5.0 using acetic acid. The temperature of the bath was increased at a rate of 5 °C/min. The sealed bowls were then rotated in a closed bath containing ethylene glycol until the treatment temperature reached 50 °C and the material : liquor ratio (M: L) was 1:15. After 40 min (treatment time), the enzymatic treatment was then terminated by raising the pH to 10.0 using Na_2_CO_3_. Samples were then removed from the bath, rinsed repeatedly with distilled hot and cold water, and then the treated fabric samples were allowed to dry in the open air. The extent of biodegradation was estimated from the weight loss (WL) of the fabric samples based on the following equation:

$${\rm WL (\%) = [W_{1} - W_{2} / W_{1}] \times\:100}$$

Where: W_1_ and W_2_ are the weights of the samples before and after enzymatic treatments.

### Carboxylic content

Carboxylic content was determined for both pretreated and treated samples by DBD plasma and alkali^25^.

### SEM and FTIR investigation

The characterization of fabric morphology was performed by scanning electron microscope (SEM, JEOL-Model JSM T2O) as previously described in literature^24^. Electron Dispersion Emission X-ray (EDX) mode was applied for the elemental composition analysis. Gold layer was deposited on the samples before analysis. The chemical structure of parent and pectinase-treated PET/C samples was determined using the Fourier transformation infrared (FTIR) spectrometer, model NEXUS 670, NICOLET USA. The measurements were carried out in spectral range from 4000 to 500 cm^− 1^. Reflection percentage measurement technique was applied (R %) to all investigated samples.

### Statistical Analysis

Data were analyzed with the help of SPSS 9.0 and the results were given as mean ± SD of three separate experiments replicates. The mean comparison between different evaluated parameters was performed using ANOVA one-way analysis of variance^26^. Statistical significance was defined when *p*< 0.05.

## Results and Discussion

### Primary and secondary Screening for pectinase production

**Fig. 1:**
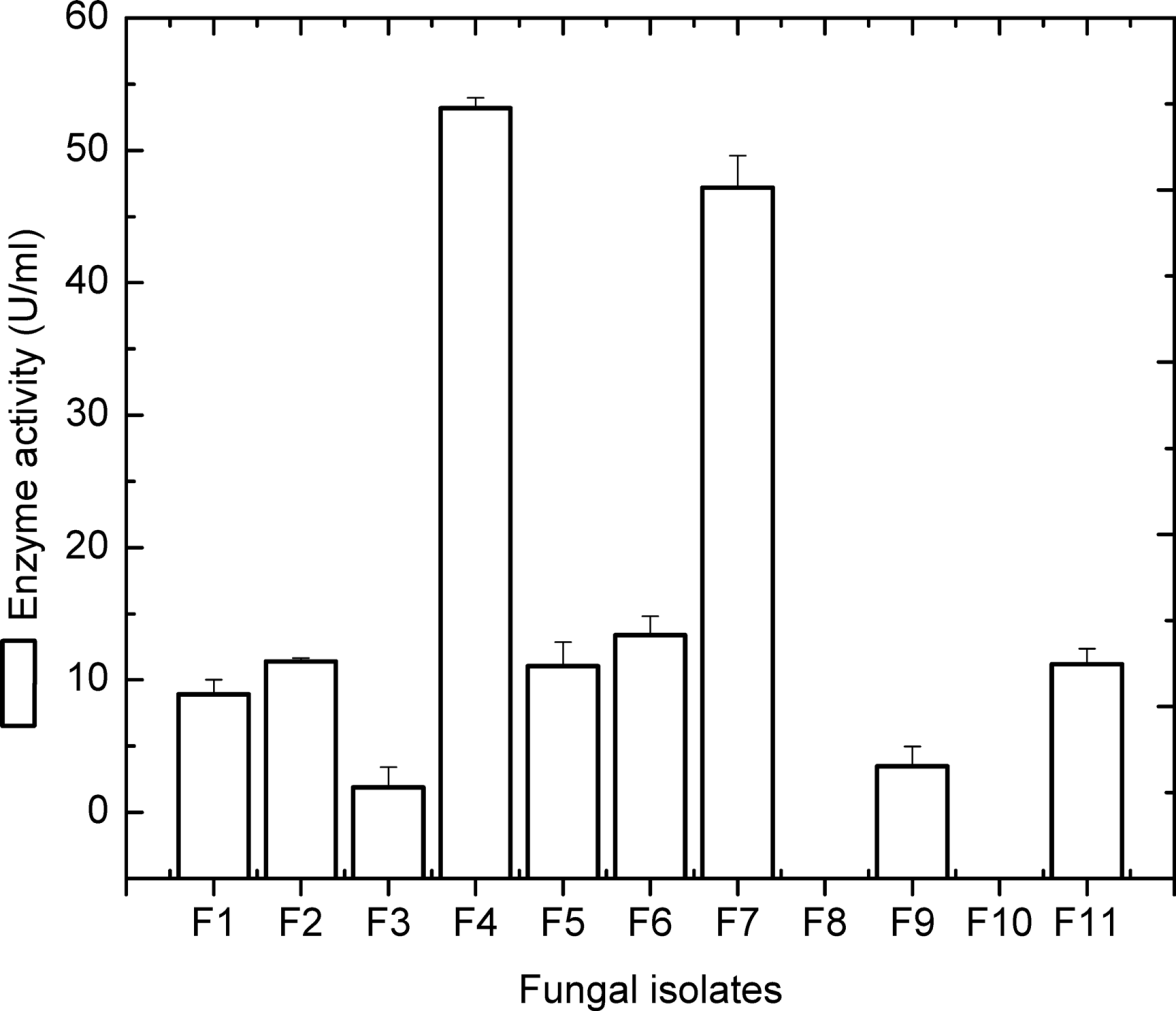
Primary screening of fungi from soil samples for pectinase production.

**Fig. 2:**
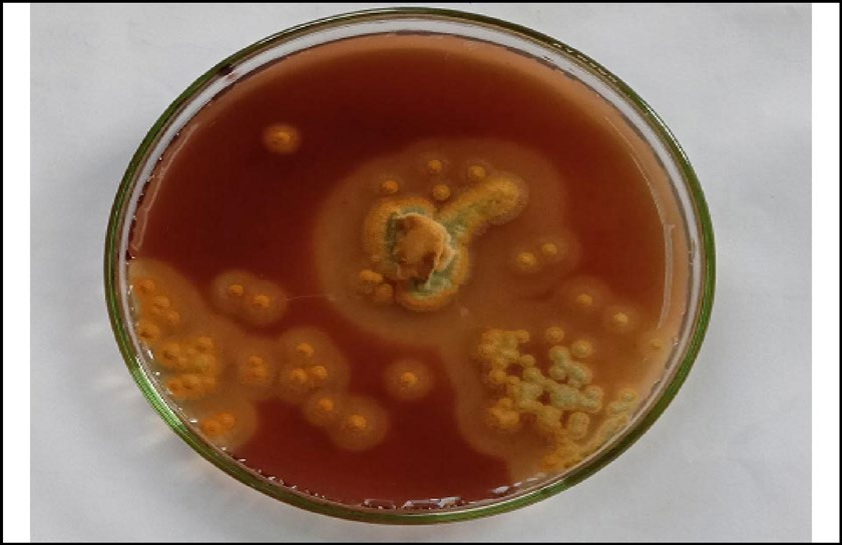
Confirmation of pectinase production by the most active isolates.

**Fig. 3:**
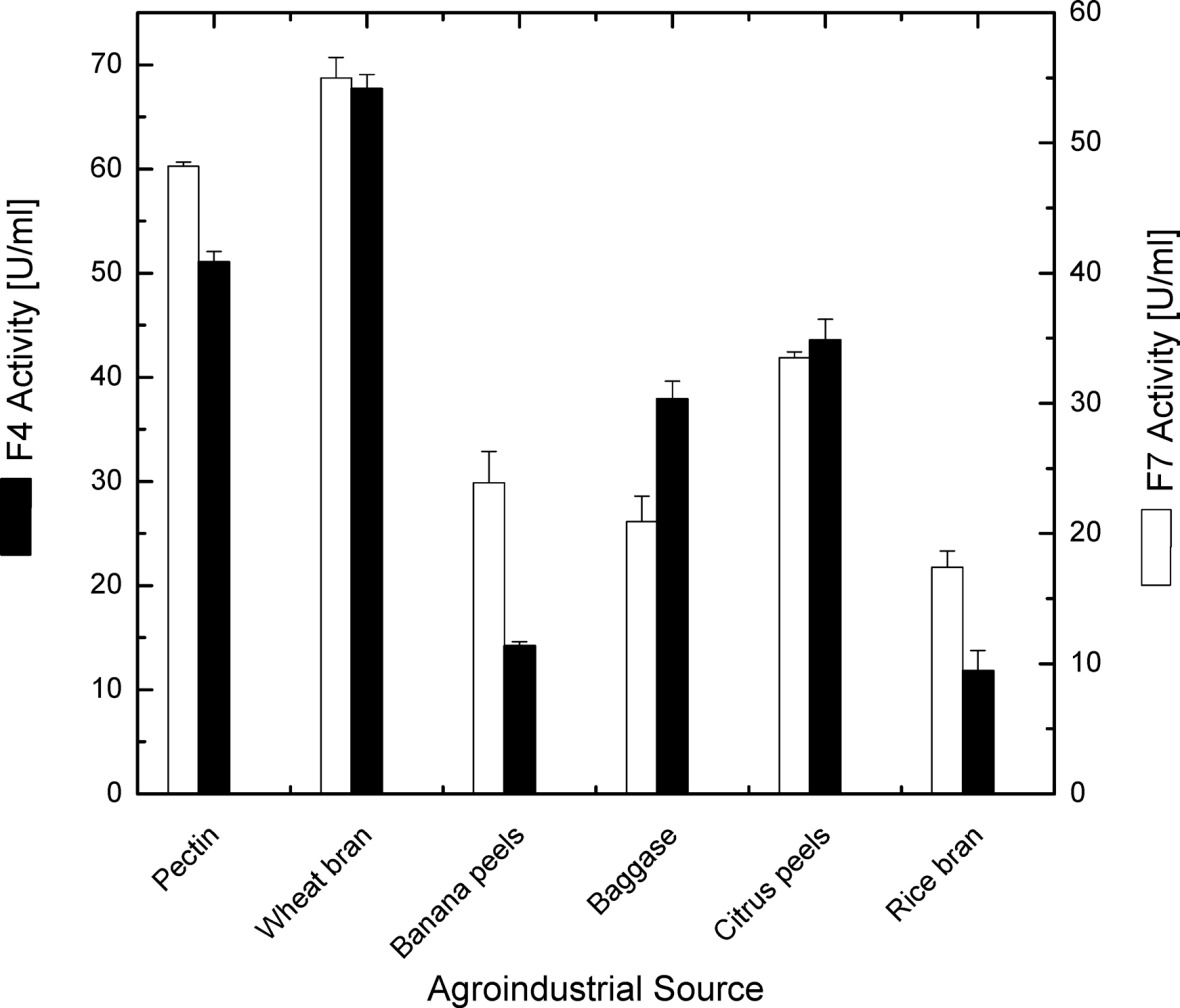
Effect of different agroindustrial substrates on pectinase production.

Among eleven fungal isolates (F1-F11) that had been purified from soil and screened for pectinase activity (Fig. 1). It can be seen that only two strains (F4 and F7) showed promising positive results, which were confirmed by the iodine test. Figure 2 showed clear inhibition zone of the enzyme producing fungi. On the other hand, Fig. 3 revealed that F4 isolate was the most active strain (67.7 U/ml) in degrading wheat bran substrate. Therefore, isolate F4 was chosen for further investigation.

#### Optimization of fermentation medium

**Fig. 4:**
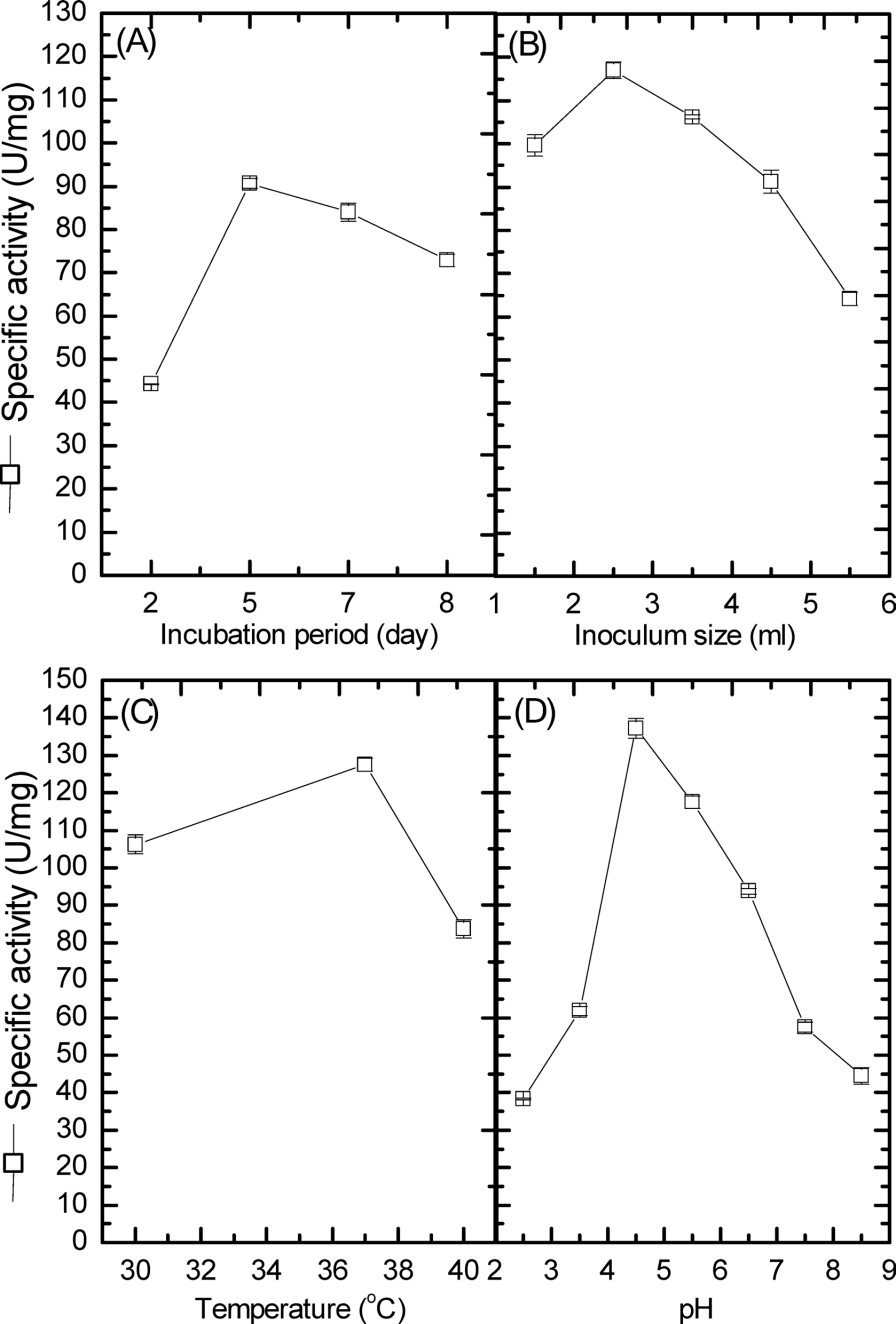
Effect of different cultivation conditions on the production of pectinase.

For optimal enzyme production, results revealed that the highest specific activity (90.7 U/mg) was obtained after 5 days of incubation (Fig. 4 A). Furthermore, the use of 2.5 ml spore suspension (4⋅10^8^ spores/ml) and 37 °C as incubation temperature were the most favorable conditions allowing maximal specific enzyme yield (128 U/mg) as shown in Fig. 4B, C. Moreover, it was found that pH 4.5 was the most suitable for optimal specific enzyme activity, which recorded 138 U/mg (Fig. 4D).

**Fig. 5:**
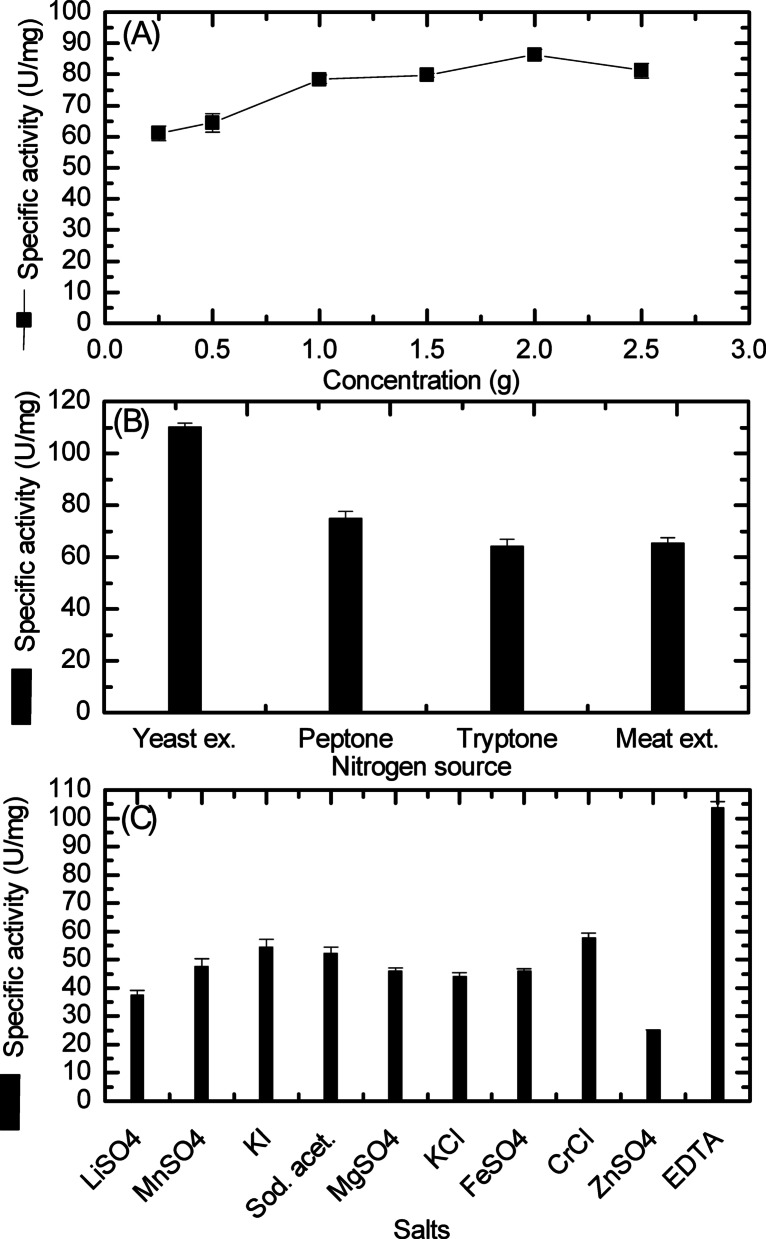
Effect of different medium compositions on the production of pectinase.

These results were in accordance with those obtained by Jalil and Ibrahim^27^. They reported that the highest pectinase production by *Aspergillus niger* LFP-1 under submerged fermentation was obtained at pH ranging from 3.5 to 4.5. They concluded that at this pH range, 90% of the pectinase activity can be retaned. By investigating the effect of different concentrations of wheat bran, results illustrated in Fig. 5 A showed that maximal specific activity of 68 U/mg was obtained upon using 2.0 g of the substrate. Furthermore, investigating the effect of different nitrogen sources on the yield of pectinase enzyme proved that yeast extract (0.5 g) was the most suitable nitrogen source allowing maximal enzyme production (Fig. 5B). These results are in accordance with those reported by Shrestha et al.^28^ and Kaur et al.^29^. They attribured their results to the stimulatory effects of yeast extract on pectinase production and secrtion. On the other hand, supplementation of the fermentation medium with 2 g/l EDTA promoted the highest pectinase production enzyme (104 U/mg) as illustrated in Fig. 5C. EDTA enhances pectinase activity by chelating divalent metal ions (e.g., Ca²⁺) that stabilize the pectin structure in plant cell walls that increases substrate accessibility and facilitates the activity of the enzyme, it may also prevent the inhibition of the enzyme by metals. Similar observation was reported by Oumer and Abate^30^, who found that EDTA enhanced pectinase activity with an increase in the obtained relative activity by about 165.3%.

#### Partial precipitation of Pectinase enzyme

A marked improvement in enzyme purity was obtained by the use of 70% acetone for precipitation of pectinase during a partial purification process Table (1). The specific activity of the culture filtrate was 20 U/mg, which increased to 51.7 U/mg after fractionation with 70% acetone, corresponding to a 2.6-fold purification. This increase in specific activity is due to the removing of impurities and other non-enzymatic proteins present in the culture filtrate^27^ Table 1.

**Table 1:** Partial purification of pectinase using 70% acetone precipitation.

Step	Total activity (U)	Total protein (mg)	Specific activity (U/mg)	Purification fold	Yield %
Culture filtrate	397	19.85	20	1	100
70% Acetone fraction	243	4.7	51.7	2.6	61

### Characterization of the partially purified enzyme

#### pH stability

**Fig. 6:**
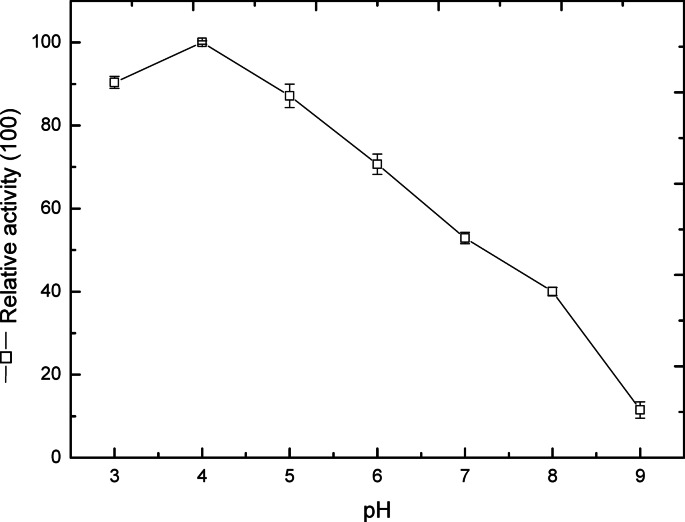
Effect of reaction pH on pectinase activity.

**Fig. 7:**
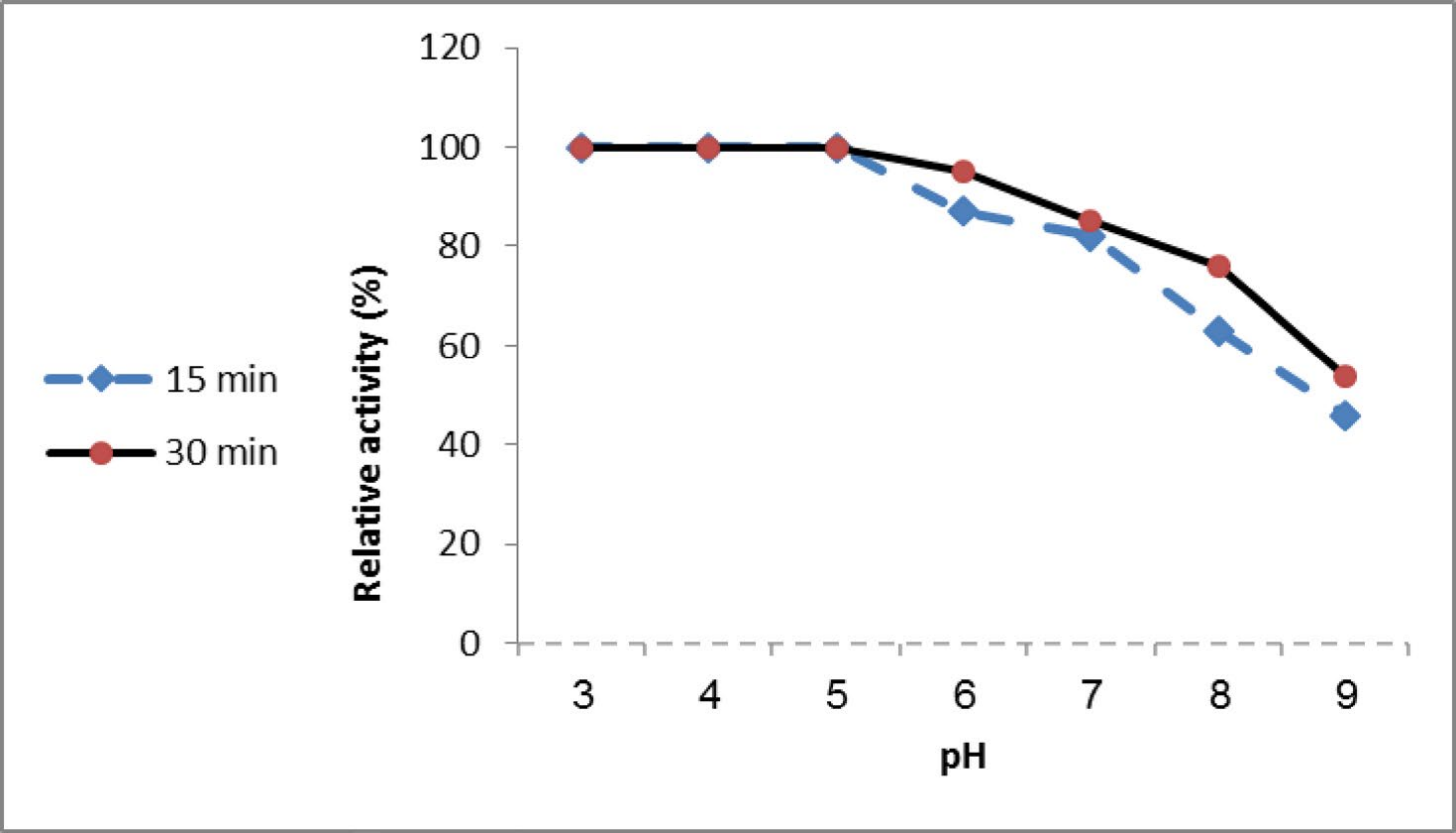
pH stability of pectin degrading enzyme.

Results in Fig. 6 revealed maximal relative activity at pH 4. Moreover, pH stability test proved that the enzyme can retain its complete activity at acidic pH conditions (3.0–5.0). These results are in accordance with those previously obtained by Ahmed and Sohail^31^, who found that pectinase was more active towards acidic pH ranges (from 3.0 to 5.5) and that the enzyme exhibited slight loss in its activity at neutral to slightly alkaline pH values (7.0–8.0). On the other side, pectinase activity decreased by 50% at alkaline medium at pH 9 (Fig. 7). Accordingly, it can be concluded that the produced enzyme showed more stability and activity at lower alkaline and higher acidic medium conditions.

#### Thermal stability and kinetic study of the enzyme

**Fig. 8:**
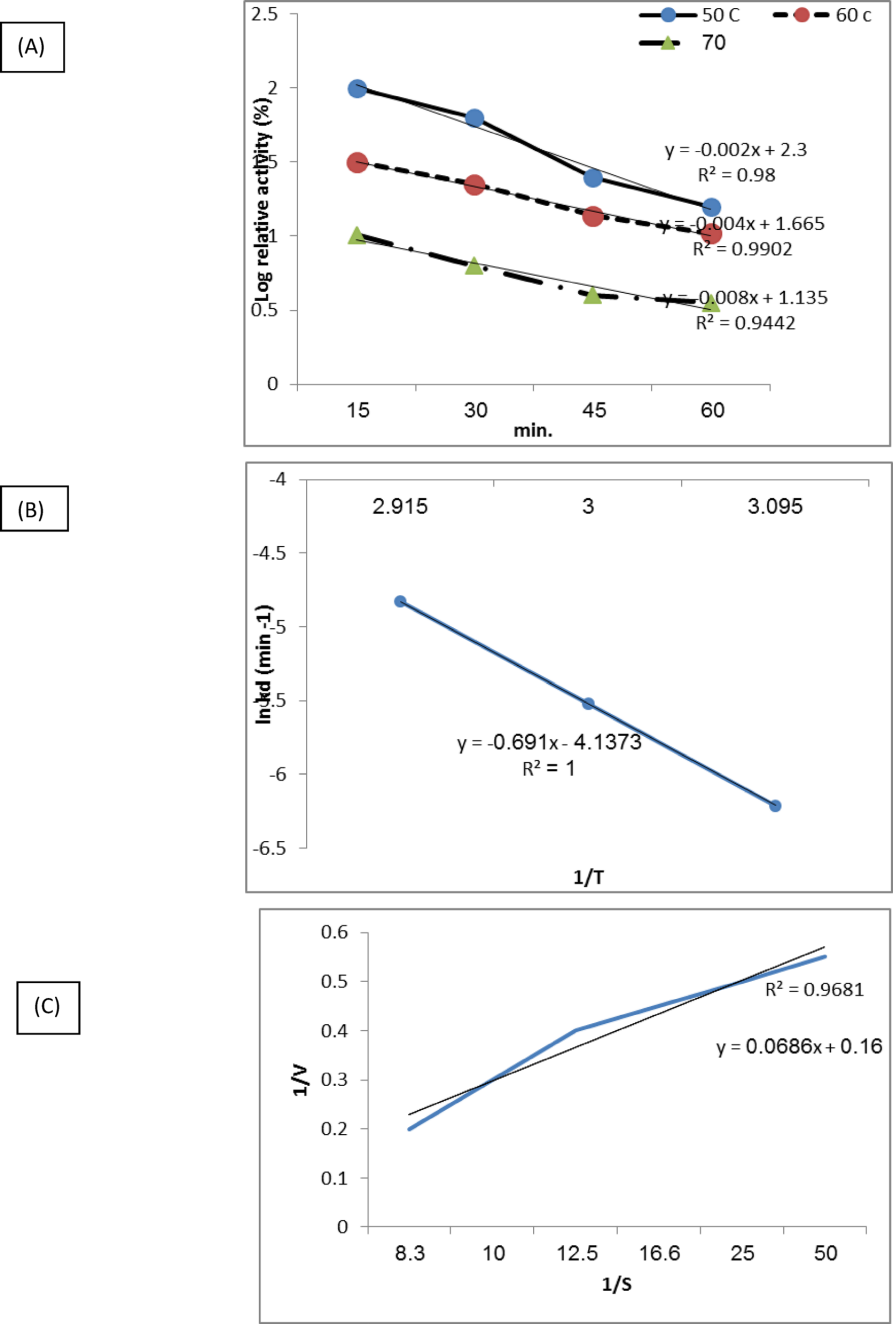
Investigated thermodynamic parameters: (A) First order reaction of thermal deactivation energy of pectinase; (B) Arrhenius plot for investigation of energy of activation for denaturation free (Ed); (C) Lineweaver-Burk plots to measure Michaelis Menten constant (K_m_) and maximum reaction rate (V_max_) values of pectinase.

Thermodynamic activation parameters (ΔH, ΔS) were measured in order to evaluate the effect of temperature on enzyme stability. A wide range of incubation temperatures was investigated for its effect on pectinase productivity. Results showed that the enzyme was most active at 50 °C and that further temperature increase resulted in the decrease in enzyme activity, thus showing higher maintenance of structural integrity of the enzyme as well as increase in the inactivation rate constant (K_d_) as presented in Fig. 8A. The same results were obtained by Ahmed et al.^32^, who reported that some thermo-stable strains can tolerate higher temperature ranges from 60 to 65 °C and produce active pectinase enzymes. They explained these effects due to differences in genetic structure of different isolated strains.

Activation energy of denaturation energy (E_d_) was calculated using Arrhenius plot as 126 KJ/mol^− 1^. This value suggests that a considerable energy is required for the unfolding process of the enzyme, thus confirming the thermal stability of the enzyme which is advantageous for textile industry (Fig. 8B). Half life time (t_1/2_) is the time required for the initial concentration of the enzyme to decrease to one half of its initial value. Since t_1/2_ and D-value decrease with increasing temperature, this confirms that the reaction rate is decreased with increasing temperature which confirms that the deactivation of the enzyme is temperature-dependent. On the other hand, ΔH is identified as the change in enthalpy, which is associated with changes in heat of a reaction in which exothermic reactions have high enthalpy and release heat.

When ΔH values were calculated over temperature range (50–70 °C), higher values reflected the high stability of the enzyme as it needs high temperature for its denaturation, thus confirming structural stability of the enzyme during industrial processes. Since the entropy (ΔS) is defined as the degree of randomness or the disorder of the system. The small negative value of the entropy indicates that there is no orientation of the solvent molecules (aqueous) around the product and a decrease of the disorder during the formation of the enzyme. This may confirm that the product is a neutral molecule (Table 2).

1 $$\:\mathrm{t}1/2\:=\frac{\mathrm{L}\mathrm{n}2}{\mathrm{k}\mathrm{d}}$$

where t_1/2_ is the half life time, Kd is the deactivation energy.

2 $$\:\mathrm{D}\:=\frac{\mathrm{L}\mathrm{n}10}{\mathrm{k}\mathrm{d}}$$

where D value is Decimal reduction time.

3 $$\:\mathrm{S}\mathrm{l}\mathrm{o}\mathrm{p}\mathrm{e}\:=\:\frac{\mathrm{E}\mathrm{d}}{\mathrm{R}}$$

where Ed is the activation energy.

ΔH° = Ed – RT(4).

where ΔH° is the enthalpy, R is the general gas constant, T is the temperature in Kelvin.

5 $$\:{\Delta\:}\mathrm{G}^\circ\:\:=-\:\mathrm{R}\mathrm{T}\:.\:\:\:\mathrm{ln}\frac{(\mathrm{k}\mathrm{d}\:.\:\:\mathrm{h})}{{\mathrm{k}}_{\mathrm{B}}\:.\:\:\mathrm{T}}$$

where ΔG° is Gibbs free energy change, h is Planck’s constant and k_B_ is Boltzmann constant.

6 $$\:{\Delta\:}\mathrm{S}^\circ\:\:=\frac{{\Delta\:}{\mathrm{H}}^{\mathrm{o}}-\:{\Delta\:}{\mathrm{G}}^{\mathrm{o}}}{\:\mathrm{T}}$$

where ΔS°is the change in Entropy.

**Table 2:** Thermodynamic parameters of pectinase enzyme.

Thermodynamic parameters	Pectinase enzyme (U/ml)
Ed (KJ.mol^− 1^)	126
t_1/2_ (min) 50 °C 60 °C 70 °C	347 173 87
D-value (min) 50 °C 60 °C 70 °C	1151 576 288
ΔHº (kJ.mol^− 1^) 50 °C 60 °C 70 °C	123.67 123.76 123.84
ΔGº (kJ.mol^− 1^) 50 °C 60 °C 70 °C	149 158 167
ΔSº (J·mol⁻¹·K⁻¹) 50 °C 60 °C 70 °C	−78 −103 −126

#### Effect of substrate concentration on enzyme activity

The kinetic parameters; K_m_ and V_max_ are essential to investigate the catalytic efficiency of the enzyme and were calculated by incubating the enzyme with different concentrations of substrate; the results were drawn as a graph of reaction rate (V) against substrate concentration (S). K_m_ is the binding rate of the enzyme with the substrate, while V_max_ is the catalytic activity which estimates the rate of conversion of substrate to product^33^. As illustrated in Fig. 8 C, K_m_ and V_max_ values were 0.4 and 6.25 respectively. These values indicate that the rate of binding between the enzyme and the substrate was fast and done in a short time.

## Molecular identification of fungal strains

**Fig. 9:**
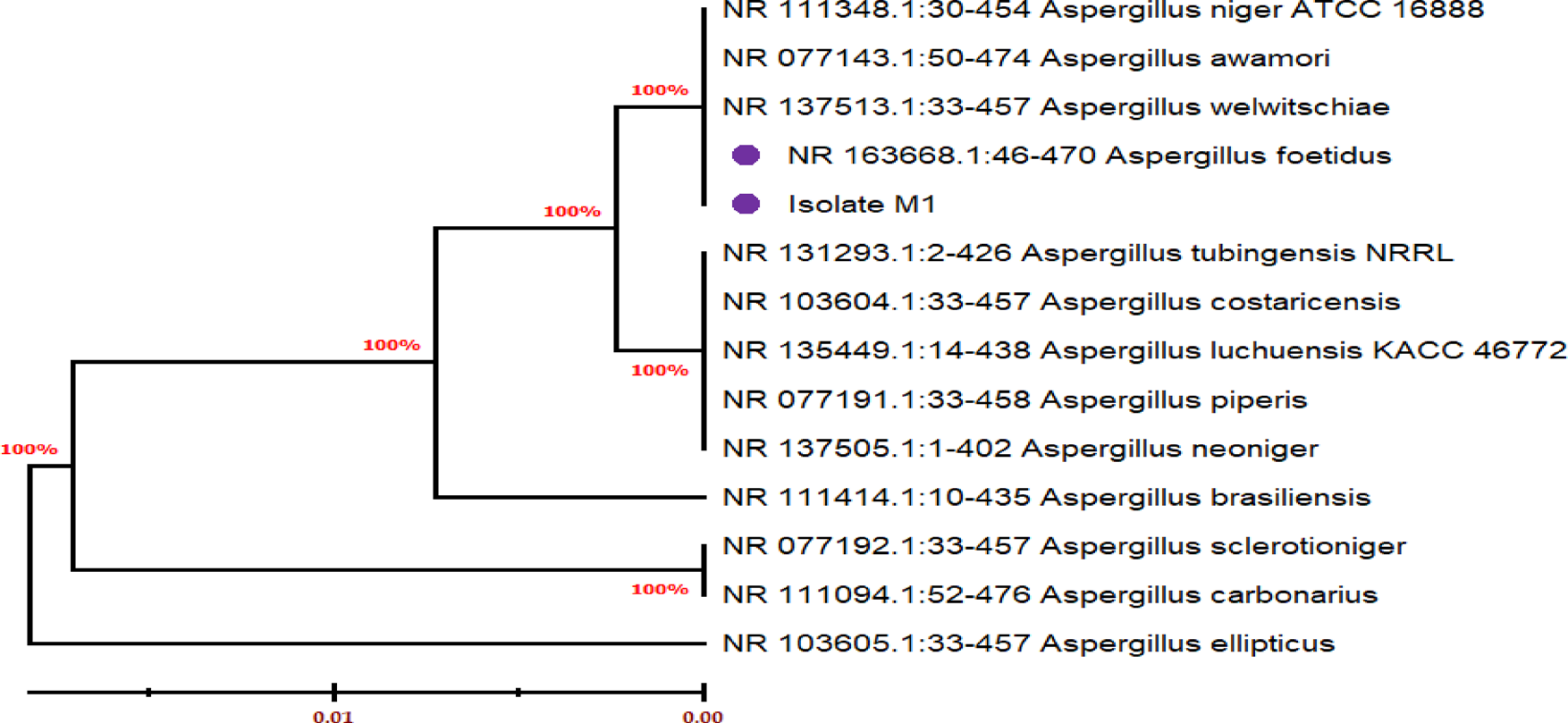
Phylogenetic tree based on partial ITS sequences, showing the relationship between isolate No (M1) Aspergillus foetidus and other species belong to the genus Aspergillus. The tree was constructed using the MEGA11 and neighbor-joining method.

The results in Fig. 9 confirmed that the active pectinase producer fungus was closely related to *Aspergillus foetidus* strain with the Accession number (NR_163668.1).

## Characterization of PET Fabrics Loaded with ZnO NPs

### Carboxylic content

PET/C blended fabrics were initially treated by pectinase enzyme, before loading with ZnO NPs. It has been reported that the biological treatment led to a significant increase of OH and COOH groups on the surface of polyester fibers^34^. These results were experimentally confirmed by the determination of functional surface groups in PET fabrics before and after the activation step. Table 2 clearly showed that surface activation increased carboxylic content in PET fabrics. The treatment brings about outstanding increase in carboxylic content from 3.95 to 38.5 meq/100 gr to PET/C blended fabrics.

#### Wettability (drop test)

**Fig. 10:**
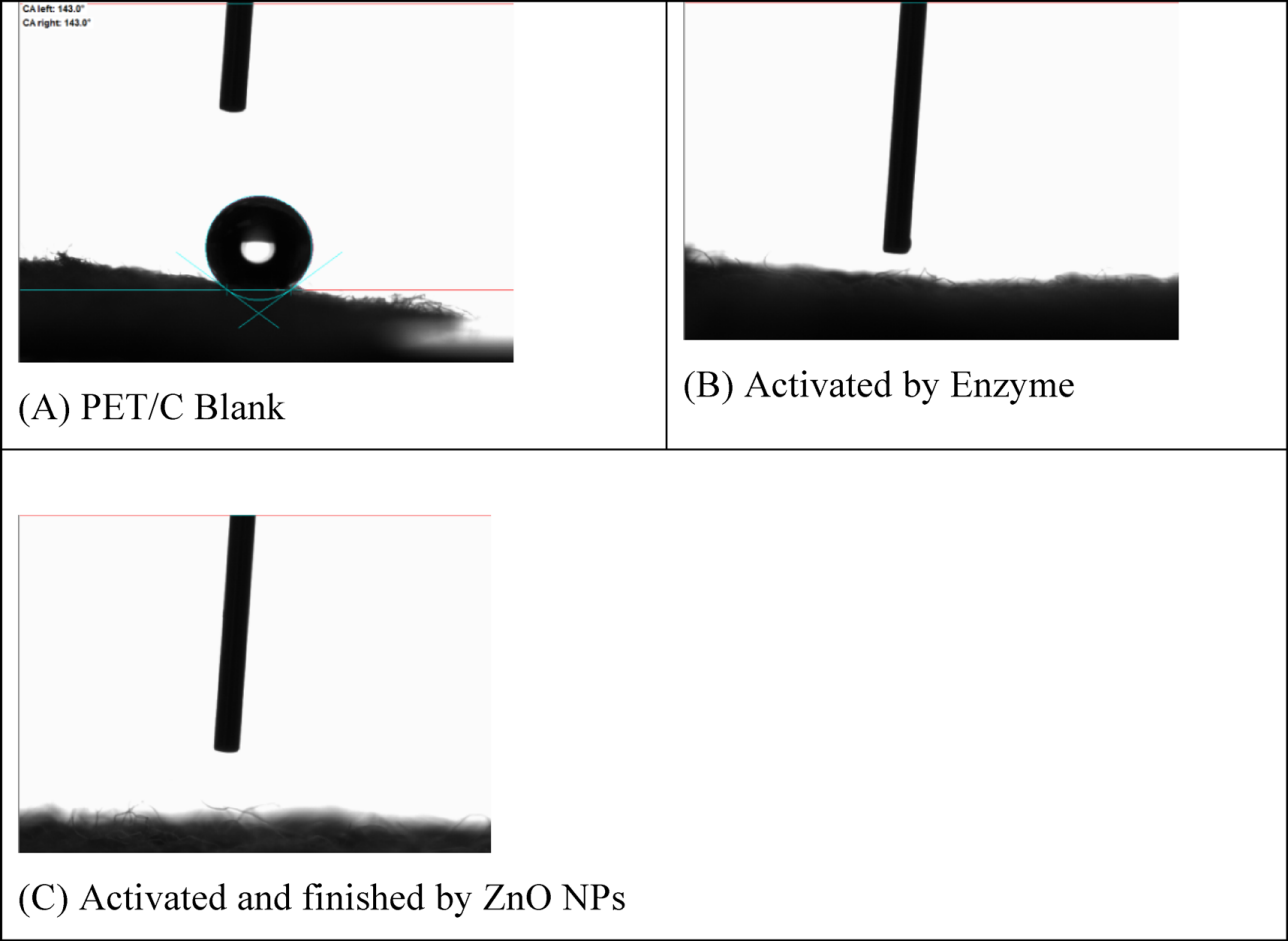
Effect of pectinase treatment on the wettability of PET/C blended fabric.

Wettability (drop test) for PET/C blended fabric before and after treatment with pectinase was evaluated by using Contact angle OCA 15EC model Company of Data Physics Instrument Gmbh. Figures (10) shows the effect of enzymatic treatment on the PET/C fabric’s wettability after pectin removal. Image (A) shows that the fabric is hydrophobic due to the presence of pectin in the cotton component and also the presence of starch (Sizing agent). The image (B) shows the effect of pectinase treatment on wettability, and that the fabric becomes hydrophilic after the removal of pectin and the sizing agent. The image (C) shows that the pectinase treated PET/C blended fabric, and loaded with ZnO NPs is still retains its wwttability properties (hydrophilic).

### Tensile strength properties

In the tensile test, tensile linearity (LT), tensile energy (WT), tensile resilience (RT), and extensibility (EMT) have been measured. Results represented in Table 3 show the average results of tensile properties for both parent PET/C blended and activated fabrics with pectinase enzyme. Applying paired comparison t test, a minor difference between tensile energy was found between the parent and activated fabrics in warp direction. Tensile energy WT increased after activation by pectinase in warp direction which mean higher stretch-ability. Also a significant difference in tensile extensibility (EMT) in warp direction was found. Extensibility EMT increased after activation in warp direction. No significant difference was found in weft direction for both previous properties. Also, a small reduction between tensile recovery (RT) was determined before and after activation of PET/C blended fabric with pectinase in both directions.

**Table 3:** Tensile properties of parent and treated PET/C blended fabric.

Tensile Parameters	Unit	Warp	Weft	Mean
*Parent PET/C blended fabric*				
Extensibility (EM)	[%]	2.84	3.40	3.12
Linearity of load/extension curve (LT)	[-]	0.793	0.821	0.807
Tensile energy (WT)	[gf×cm/cm^2^]	560	6.99	6.30
Tensile resilience (RT)	[%]	72.86	70.57	71.72
*Treated PET/C blended fabric*				
Extensibility (EM)	[%]	4.48	3.81	4.15
Linearity of load/extension curve (LT)	[-]	0.748	0.770	0.759
Tensile energy (WT)	[gf×cm/cm^2^]	8.31	7.37	7.84
Tensile resilience (RT)	[%]	63.68	67.31	65.50

#### SEM

**Fig. 11:**
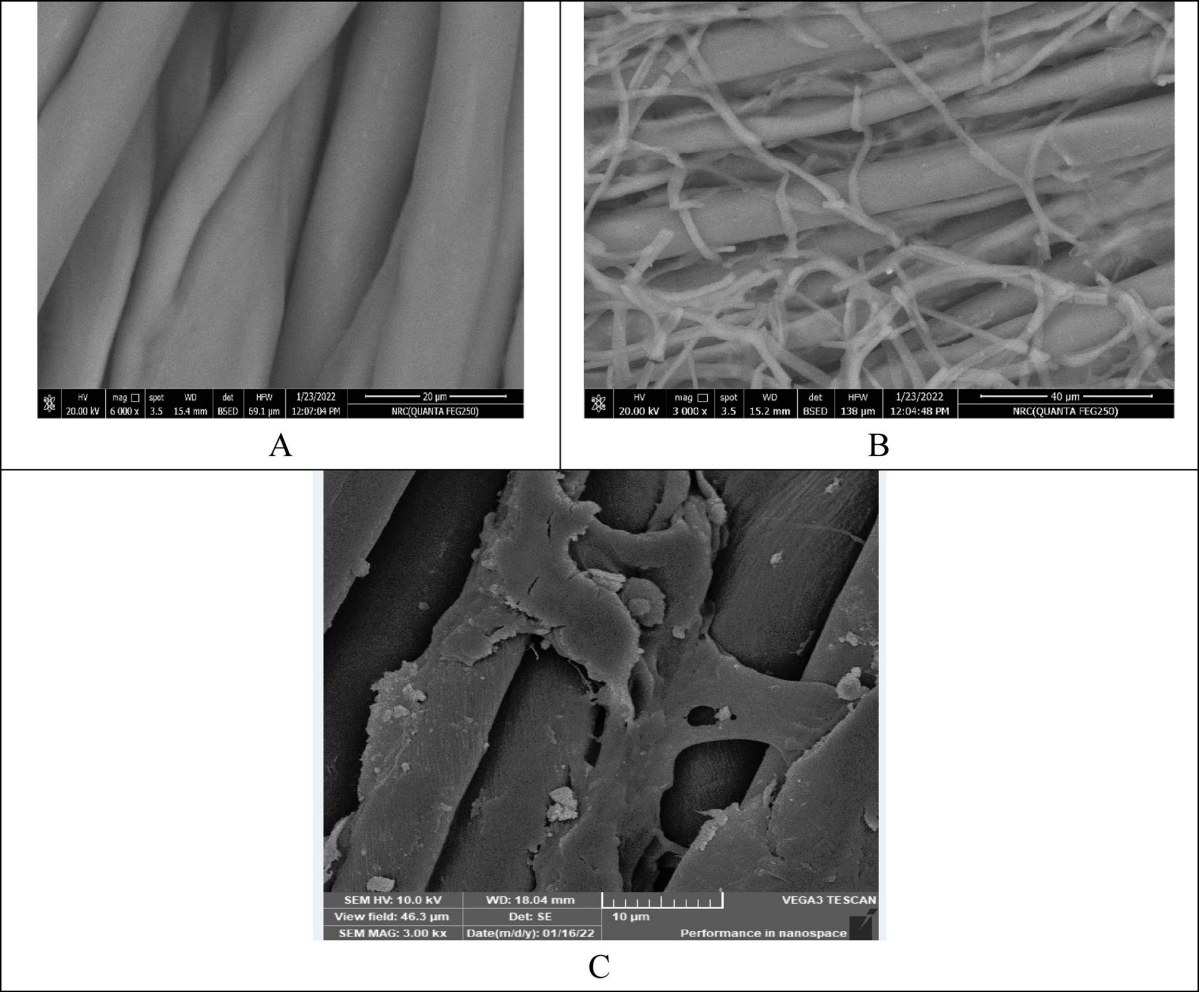
SEM images of [A] Parent PET/C Fabric; [B and C]PET/C Fabric Treated with Pectinase enzyme (3000X).

From Fig. 11; Table 4 it can be observed that, the PET/C blended substrate was strongly affected by pectinase treatment. Weight loss reached about ~ 4% indicating successful pectin hydrolysis. It is well known that, pectin found in cellulosic fibers such as cotton, inhibits the absorption of aqueous solutions. Therefore, when fabrics are treated with pectin-degrading enzymes, the fabrics become easier to absorb and access the terminal carboxyl groups present in polyester. This is conjugated with increasing the number of COOH end groups of ester molecules involved in the reaction and bonding with the ZnO NPs. In addition to that, the pectinase treatment leads to increasing the number of hydroxyl groups reacting with ZnO NPs where pectinase attacks the glucoside molecules and breaks the ether bonds along the cellulosic chains^35^. These results were also confirmed by the increase in carboxylic content after treatment process (Table 4). Accordingly, increasing surface functionality on cotton blend substrate would facilitate the interaction with textile finishing agents as metal oxide nanoparticles. SEM in Fig. 10 for cotton blend substrate before and after enzymatic hydrolysis confirmed the strong pectinase effect on the surface of cotton blend fibers. Significantly, the fibers became de-bundled and ruptured with enzymatic treatment, indicating successful hydrolysis of glucoside and ether bonds along the cellulosic chains and between the fibers as well as bundles.

**Table 4:** Effect of the Pectinase Treatment on the Amount of Carboxylic Content of PET/C Blend Fabrics.

Fabrics	Weight loss (%)	Carboxylic content (meq/100 gram Fabric)
PET (Parent)	0.0	3.30
PET→ Pectinase enzyme	3.90	38.5

Enzymatic Treatment Conditions: [Pectinase]: 1.0%, pH = 5.0, Time, 40 min, Temperature, 45 °C, M: L, 1:15.

**FTIR**.

**Fig. 12:**
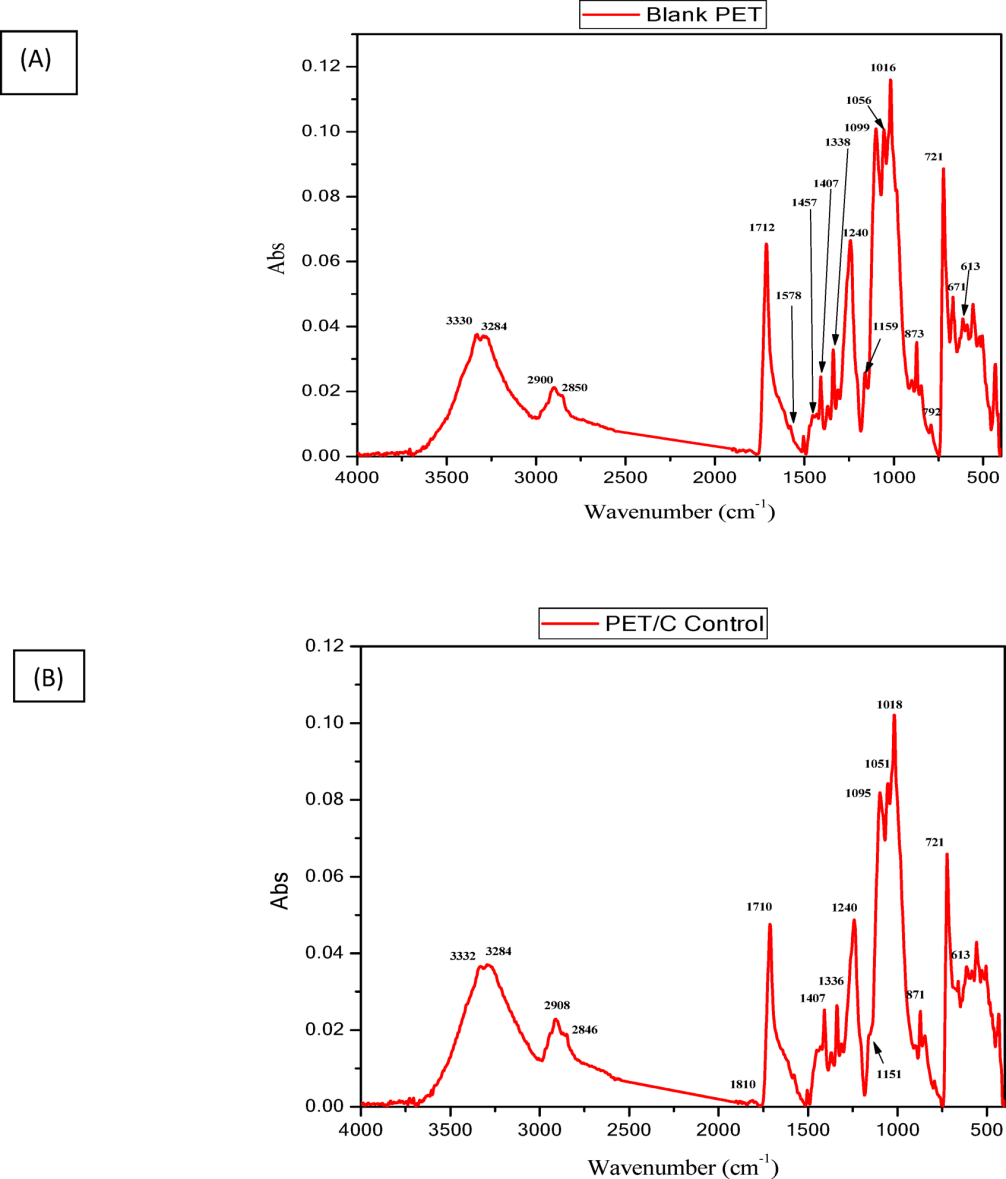
FTIR spectra of PTE/C blended fabric; (A) Parent and (B) pectinase-treated.

FTIR spectra of parent and pectinase-activated PET/C blended fabrics were summarized in Table 5 and shown in Fig. 12 A and B, respectively. The spectrum of parent PET/C parent fabrics (Fig. 12 A) showed the functional groups of un-activated fabric at characteristics signals: 1712, 3284 and 2900 cm^− 1^ which were typical to those of > C = O, OH and CH stretching, respectively. On the other hand, the spectra of pectinase-activated fabrics (Fig. 12B) exhibited additional characteristics signals ranging from 1600 to 1500 cm^− 1^ for carboxylate anions (COO¯). Similar findings were reported by Al-Balakocy et al.^36^. It can be seen that, treatment of PET/C fabrics with pectinase do not cause any remarkable change in the spectrum (Fig. 12) except for changes in the relative intensities of the functional groups OH and COOH. This indicates that, active groups have been introduced onto PET/C fabrics surfaces. These findings were confirmed by carboxylic content measurement as previously discussed in Table 4.

**Table 5:** FTIR spectra of parent and pectinase-activiated PTE/C blended fabrics.

Peak position (Cm^− 1^)		Assignment
Before	After	
3284	3284	-OH, Stretching
2900	2908	C-H, Stretching
2850	2846	CH2,, Stretching vibration of symmetric
1712	1810	> C = O, Ester carbonyl
1338	1407	CH2, Alkane
-	1500, 1600	COO¯, Carboxylate
1240	1240	C-O, Carboxylic acid
1159	1151	C-O-C asymmetric stretching
1099, 1016	1095, 1018	O = C-O-C ester
671, 613	671, 613	C–OH bending

Enzymatic Treatment Conditions: [Pectinase]: 1.0%, pH = 5.0, Time, 40 min, Temperature, 45 °C, M: L, 1:15.

## Conclusions

Due to their numerous advantages, attention has been paid to the use of biological agents and methods as potential alternatives to chemicals in biotechnological industrial processes. The current work aimed at investigating the possibility of pectinase enzymes produced from locally isolated strain to replace chemical agents, which are generally used in textile industries. This will not only avoid the harmful effects of chemical agents on the fabric structure and properties, but also reduces environmental pollution effects, and thus the textile process can be eco-friendly. The most potent isolated fungal strain was able to produce pectinase under optimized medium composition and conditions. Wheat bran proved to be the most suitable agro-industrial waste allowing highest enzymatic productivity, thus highlighting the possibility of designing cost-effective production bioprocess. Afterwards, pectinase was characterized, precipitated and thermodynamically investigated. It can be seen that lower enzymatic activation energy and higher inactivation constant as well as deducted kinetic parameters suggest the potential application of the produced enzyme as a biocatalyst in textile industry. Characterization of PET/C blended fabrics treated by pectinase proved the efficiency of pectinase application in fabric treatment. This potential was proven through the loss of fiber weight (~ 4%) indicating successful hydrolysis of pectin, which was also confirmed through carboxylic content analysis. Enzyme-treated fiber exhibited micro-graphical alterations showing de-bundled and ruptured fibers. Furthermore, treated blend fibers showed increased surface functionality facilitating the interaction with textile finishing agents such as metal oxide nanoparticles to impart smart properties.

## Data Availability

BLAST result of ITS gene sequence of isolate No (2) Aspergillus foetidus strain 2 have been deposited in the Gene Bank under the Accession Number NR_163668.1.
